# InterDILI: interpretable prediction of drug-induced liver injury through permutation feature importance and attention mechanism

**DOI:** 10.1186/s13321-023-00796-8

**Published:** 2024-01-03

**Authors:** Soyeon Lee, Sunyong Yoo

**Affiliations:** 1 Department of ICT Convergence System Engineering, Chonnam National University, Gwangju, 61186 Republic of Korea; 2 Division of Bioresources Bank, Honam National Institute of Biological Resources, Mokpo, 58762 Republic of Korea

**Keywords:** Attention mechanism, Drug-induced liver injury, Feature importance, Hepatotoxicity, In silico prediction

## Abstract

Safety is one of the important factors constraining the distribution of clinical drugs on the market. Drug-induced liver injury (DILI) is the leading cause of safety problems produced by drug side effects. Therefore, the DILI risk of approved drugs and potential drug candidates should be assessed. Currently, in vivo and in vitro methods are used to test DILI risk, but both methods are labor-intensive, time-consuming, and expensive. To overcome these problems, many in silico methods for DILI prediction have been suggested. Previous studies have shown that DILI prediction models can be utilized as prescreening tools, and they achieved a good performance. However, there are still limitations in interpreting the prediction results. Therefore, this study focused on interpreting the model prediction to analyze which features could potentially cause DILI. For this, five publicly available datasets were collected to train and test the model. Then, various machine learning methods were applied using substructure and physicochemical descriptors as inputs and the DILI label as the output. The interpretation of feature importance was analyzed by recognizing the following general-to-specific patterns: (i) identifying general important features of the overall DILI predictions, and (ii) highlighting specific molecular substructures which were highly related to the DILI prediction for each compound. The results indicated that the model not only captured the previously known properties to be related to DILI but also proposed a new DILI potential substructural of physicochemical properties. The models for the DILI prediction achieved an area under the receiver operating characteristic (AUROC) of 0.88–0.97 and an area under the Precision-Recall curve (AUPRC) of 0.81–0.95. From this, we hope the proposed models can help identify the potential DILI risk of drug candidates at an early stage and offer valuable insights for drug development.

**Supplementary Information:**

The online version contains supplementary material available at 10.1186/s13321-023-00796-8.

## Introduction

Drug-induced liver injury (DILI) refers to injuries caused to the liver by various supplements, herbs, medications, or other chemical compounds, resulting in liver dysfunction [1, 2]. From mild abnormalities to acute liver failure, DILI can cause extensive liver damage and, in some cases, can be fatal. The estimated global annual prevalence rate of DILI was 13.9 ± 2.4 per 100,000 people [3]. DILI usually occurs by unpredictable drug reactions or idiosyncratic metabolic responses, creating difficulties in drug discovery. There have been numerous reports of adverse drug reactions and severe toxicities leading to the withdrawal of drugs from clinical studies by pharmaceutical companies [4]. The results of clinical trials show that approximately 90% of new active substances fail to gain regulatory approval due to poor efficacy and unexpected toxicity [5]. Despite being developed and marketed successfully, drugs can be withdrawn if they cause side effects. Among the types of adverse drug reactions, DILI has been the leading cause of drug withdrawal and disapproval during drug discovery in recent decades [6–8]. Therefore, early prediction and assessment of DILI are major challenges in drug development.

In general, experimental animal models are used to predict DILI. However, they are time-consuming, labor-intensive, and have poor concordance between species [9]. A previous study found that 43% of clinical toxicities were not identified in animal studies for 64 marketed drugs [10]. In vitro cellular models are limited in representing the complexities of human DILI and are ineffective for toxicity mechanisms. Several studies on DILI risk prediction using in silico methods have been proposed to alleviate these issues. In previous studies, conventional machine learning methods, such as k-nearest neighbor Bayesian modeling, random forest (RF), support vector machine (SVM), and extreme gradient boosting algorithms, have been executed to predict hepatotoxicity [11–13]. In addition, the importance of the molecular substructure of the RF was assessed using the Gini coefficient [14]. The bits in molecular fingerprints corresponded to different chemical groups, and the fingerprint with a high Gini coefficient indicated the hepatotoxic risk of the chemicals in the study [13]. A few attempts have also been made to develop deep-learning models based on molecular fingerprints [15, 16]. A convolutional neural network (CNN) based on embedded molecular fingerprint features was used for predicting DILI [16]. However, most of the studies focused on performance improvement rather than interpretation. In addition, nearly all the studies identified feature importance only for feature selection or specific models. Recently, it has been proposed to use an attention mechanism to identify structure–activity or the structure–property relationship to interpret deep learning architectures [17]. Zheng et al. predicted various chemical properties such as aqueous solubility, stability, and bioactivity.

This study applied permutation feature importance and attention mechanism to machine learning models for interpretable DILI predictions. This study recognized general-to-specific patterns, which focused on the overall importance of features in each model and how the specific molecular substructure significantly contributed to the DILI prediction for each compound. First, to achieve this, public datasets were collected. The molecular descriptors, including substructure and physicochemical properties, from the obtained compounds were calculated to predict DILI. With these features, RF, light gradient boosting machine (LGBM), logistic regression (LR), and neural network (NN) with attention models were built. Lastly, permutation feature importance for general patterns and self-attention for specific patterns were employed.

## Materials and methods

### Data collection

**Fig. 1:**
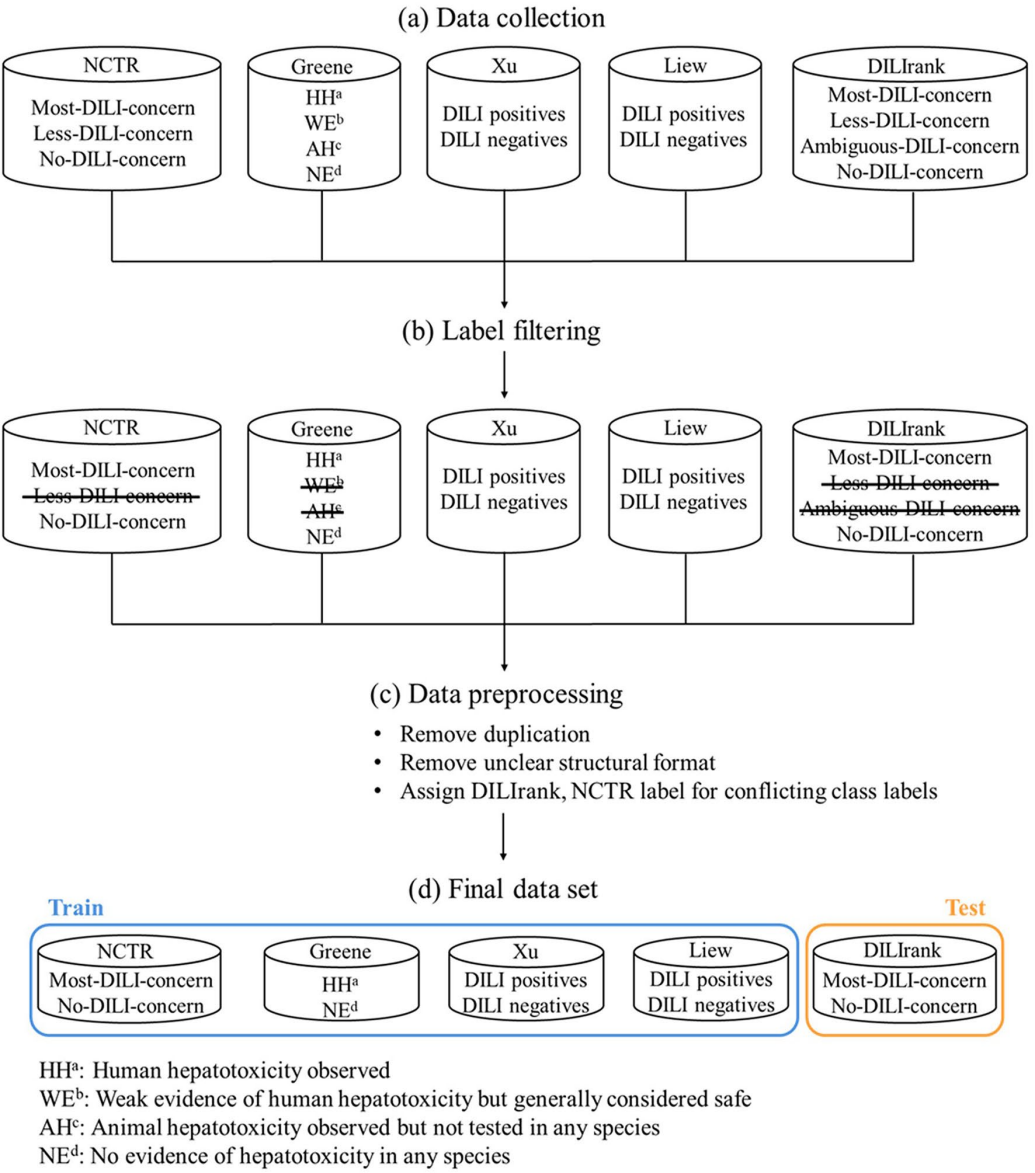
Overall data collection process. **a** First, we collected the public five datasets. **b** DILI positives and DILI negatives were selected by filtering drugs and compounds clearly related to DILI. **c** After integrating the datasets, duplicates and unclear structural formats were removed. Additionally, when the class label of compounds had a conflict, the DILIrank or NCTR data label was assigned. **d** NCTR, Greene, Xu, and Liew datasets were used as the training set and the DILIrank dataset was used as the test set

Five publicly available datasets were collected (Fig. 1a).

The first dataset was from the Food and Drug Administration’s (FDA) National Center for Toxicological Research (NCTR) has established FDA-approved drug labeling to assess the potential risks of DILI in humans [18]. The NCTR divides drugs into the following three classes: (i) drugs with a Boxed Warning and that have been withdrawn from the market are labeled “most-DILI-concern”; (ii) drugs that contain no precautions regarding DILI are labeled “no-DILI-concern”; and (iii) drugs that do not meet the other categories are labeled “less-DILI-concern.”

The second dataset was from a study by Greene et al. assigned compounds to the following four classifications associated with hepatotoxicity: (i) compounds with observed human hepatotoxicity were labeled “HH”; (ii) compounds that were generally considered safe but had weak evidence of human hepatotoxicity were labeled “WE”; (iii) compounds with observed animal hepatotoxicity but not tested on humans were labeled “AH”; and (iv) compounds that had no evidence of hepatotoxicity in any species was labeled “NE” [19].

The third dataset was from a study by Xu et al. classified drugs into the following groups using clinical data on hepatotoxicity: (i) drugs not marketed in the US, withdrawn from the market, received a black box warning from the FDA, or had more than ten clinical reports due to hepatotoxicity were labeled as “DILI positive,” drugs marketed with a hepatotoxicity warning on the label, had a well-known association to liver injury, or a Pfizer internal drug candidate whose development was ceased due to hepatotoxicity concern were also labeled as “DILI positive”; and (ii) drugs that did not meet any of the above positive criteria were labeled as “DILI negative” [20].

The fourth dataset was from a study by Liew et al. divided drugs into the following two classes: (i) drugs with transient and asymptomatic liver function abnormalities, liver function abnormalities, hepatitis, jaundice, cholestasis, fulminant hepatitis, liver failure, or fatality to the liver were labeled as “DILI positive”; and (ii) drugs not associated with any adverse hepatic effects were labeled as “DILI negative” [21].

The fifth dataset was the Drug-Induced Liver Injury Rank (DILIrank) is the largest reference drug list related to DILI in humans [22]. The DILIrank dataset is composed of FDA-approved drugs and divided into the following four classes: (i) drugs with potential to cause severe clinical outcomes are labeled as “Most-DILI-concern”; (ii) drugs that can cause liver injuries but rarely lead to severe outcomes are labeled as “Less-DILI-concern”; (iii) drugs with a low perceived risk and rare or nonexistent liver injuries are labeled as “No-DILI-concern”; and (iv) drugs with a DILI concern but without verified causality were labeled as “Ambiguous-DILI-concern drug.” This study tried to use only drugs and compounds clearly associated with DILI, thus drugs labeled as "less-DILI-concern" in the NCTR, compounds labeled as “WE” and “AH” in the study by Greene et al., and drugs labeled as “Less-DILI-concern” and “Ambiguous-DILI-concern” in the DILIrank were excluded (Fig. 1b).

These datasets from five different studies have been integrated into a single dataset. First, duplicate compounds were detected and eliminated based on PubChem’s compound identifier (CID). Then the canonical simplified molecular-input line-entry system (SMILES), a chemical notation method that represents the molecular structure as a character string, was obtained based on their CID [23]. After that, compounds with the same or unclear SMILES were eliminated. When the class labels conflicted, the label from the DILIrank or NCTR datasets, which strictly classified the drug, was assigned (Fig. 1c). Four data sets, NCTR, Greene, Xu, and Liew, were used as the training set. The DILIrank was only used as the test set (Fig. 1d). Finally, the training set consisted of 1398 compounds, of which 768 were DILI positive and 550 were DILI negative. The test set consisted of 452 compounds, of which 184 were DILI positive and 268 were DILI negative. A summary of the final dataset is shown in Table 1.

**Table 1:** Data summary of the final dataset

Category	Dataset	DILI positive	DILI negative	Total
Training (76%)	NCTR	169	179	348
	Greene	149	80	229
	Xu	12	34	46
	Liew	438	337	775
	Total	768	630	1398
Test (24%)	DILIrank	184	268	452

### Molecular descriptors

Molecular substructure and physicochemical properties were used as input features in this study. First, the SMILES structure was converted to a molecular fingerprint, a way to describe the molecular structure by converting it into a bit string. An extended-connectivity fingerprint (ECFP), one of the molecular fingerprints, was used in this study. ECFP is designed to capture the local structural features [24]. The ECFP was followed by a number indicating the number of the largest effective diameter. The number is equal to twice the number of iterations performed. This study used the ECFP6. The ECFP6 contains all the possible paths through an atom with a radius of 3 and extracted molecular substructures with a maximum width of six bonds. In addition, the ECFP transformed the molecular structures into vectors of integers in a given dimension. The higher the radius and dimension, the better the bit collisions are avoided. Lastly, the ECFP represents the presence of specific molecular substructures, making analysis results easy to interpret. For example, a “1” is assigned when the substructure exists, and a “0” is assigned when the substructure does not exist. An ECFP6 of 1024 bits was generated as the molecular descriptor for each compound in the datasets. In summary, when the radius size was set at 3, the substructures of the molecules with a radius size of 3 or less were extracted and converted into numerical identifiers by a slightly modified Morgan algorithm [25]. The identifiers of all extracted substructures were hashed into a binary vector of 1024. Consequently, the binary vector of set bits represented the molecular substructure. The mean and standard deviation of each binary vector values are displayed in Additional file 1: Fig. S1.

In addition, eight physicochemical descriptors were calculated from the SMILES, including the molecular weight (MW), octanol–water partition coefficient (ALOGP), number of hydrogen bond donors (HBD), number of hydrogen bond acceptors (HBA), polar surface area (PSA), number of rotatable bonds (ROTB), number of aromatic rings (AROM), and number of structural alerts (ALERT). Some of these physicochemical descriptors are known to be strongly related to the DILI prediction [26, 27]. As the values of the physicochemical descriptors varied significantly, they were scaled to zero mean and one variance [28]. Additional file 1: Fig. S2 shows the distribution of theses physicochemical descriptors and Additional file 1: Fig. S3 shows the distribution after applying the standard scaler to the physicochemical descriptors. All the features were calculated using an open-source cheminformatics software, RDKit [29].

### Machine learning models

The RF, LGBM, LR, and NN with attention models were constructed and optimized. The RF is an ensemble method that learns a multitude of decision trees [30]. First, 1398 bootstrap sets, a subset of the training set through sampling with replacement, were generated. Then, a decision tree was trained using the bootstrap set with randomly selected features. The process was repeated to generate a multitude of decision trees. Finally, the prediction was calculated by averaging the predictions of each decision tree. The following hyperparameters of the RF were optimized; the number of trees in the RF, the maximum number of features considered for splitting a tree, and the maximum depth in each decision tree.

LGBM is a gradient-boosting framework and a tree-based learning algorithm [31]. Decision trees have a level-wise growth strategy, whereas LGBM has a leaf-wise growth strategy with depth constraints. By selecting the leaf that was expected to reduce loss the most, the tree was grown vertically. The LGBM has the advantages of fast training speed and higher efficiency but has the risk of overfitting. The following hyperparameters of the LGBM were optimized; the max number of leaves in one tree and the number of boosting iterations. To deal with overfitting, two hyperparameters were optimized; the minimal number of data in one leaf and the maximum depth of the tree model.

LR calculates the likelihood of an event occurring based on an independent variable dataset [32]. The magnitude of the LR coefficients represents the relative importance of each independent variable in influencing the prediction. Larger coefficient values indicate a stronger impact on the prediction, while smaller coefficients suggest a weaker influence. This interpretation allows for identifying the importance of each independent variable in the prediction. The following hyperparameters of the LR were optimized; the maximum number of iterations taken and the strength of the regularization. To obtain optimal hyperparameters for each model, RandomizedSearchCV in Scikit-learn was applied [33]. RandomizedSearchCV tries random combinations of a predefined range of hyperparameter sets and searches for better models. The optimal hyperparameter sets were obtained with 50 iterations. Optimal parameters obtained from the hyperparameter tuning of each model are shown in Additional file 1: Table S1.

A self-attention mechanism was adopted to determine which parts of the molecule influenced the prediction model [34]. The attention mechanism was used to weigh the importance of features by calculating the correlation between the inputs and output. First, the vectorized structure and physicochemical descriptors were concatenated. The concatenated vectors were fed into the fully connected layer. An attention mechanism then took them as the input and output vector of weights (${W}_{att}$). Formally, the formula is:

1 $${W}_{att}= softmax\left(g\left(inp\right)\right)$$

2 $$g\left(x\right)= Wx+ b$$

where ${W}_{att}$ is the attention weight, $softmax$ is the function that normalizes the sum of the vectors to be 1, and $inp$ is the input vector, which is the concatenated vector, and where $g\left(x\right)$ is the fully connected layer without the activation function, $W$ is the weight matrix, and $b$ is the bias. The output of the $g\left(inp\right)$ function was used as the input for the $softmax$ function, and the attention weight was calculated. Then, an element-wise product of the attention weight and input vector was conducted using the formula:

3 $$v\, = \user2{ }\,W_{att} \, \odot \, inp$$

where ⨀ denotes the element-wise product and $v$ is the weighted vector. After this, $v$ was used as the input for the multilayer perceptron (MLP). Each dense layer except the last layer was applied to the ReLU activation and He initialization [35, 36]. In addition, batch normalization for the model regularization was performed and a 0.25 dropout rate was set after each layer to prevent overfitting [37, 38].

MLP contains multiple layers, and each layer is composed of multiple nodes. To obtain the optimal number of layers and nodes, a Bayesian optimization was done [39]. Bayesian optimization creates a surrogate model for the objective function and hyperparameter pairs and explores the optimal hyperparameter set through evaluations by updating the hyperparameters sequentially. The optimal number of layers and nodes is shown in Additional file 1: Table S1.

**Fig. 2:**
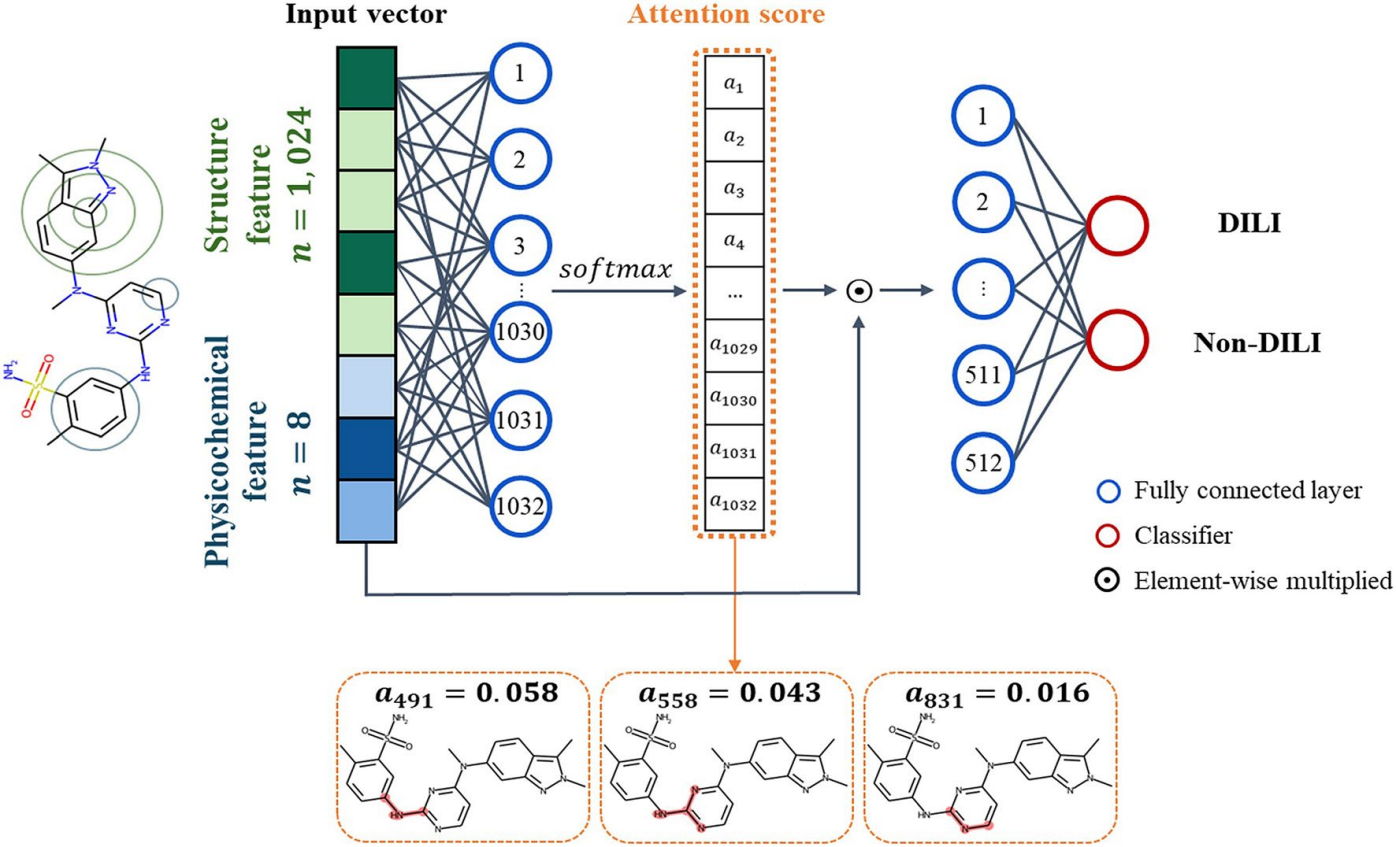
The structure of NN with attention. Physicochemical and structural descriptors were calculated from the compound. Then, both features were concatenated and took into account the fully connected layer. An attention mechanism took them as the input and output an attention weight. The attention weight was element-wise multiplied with the input vector and fed into the fully connected layer. After that, the sigmoid activation function classified the DILI label. Finally, the substructures of the molecules that influenced the model prediction were identified by analyzing the attention weight

As a result, the weighted feature vector (${\varvec{v}}$) was fed into one layer with 512 nodes, and the sigmoid as an activation in the last layer was used to predict DILI. Binary cross-entropy loss, early stopping, Adam optimizer, 100 epochs, and 32 batch size were employed [40]. The structure of NN with attention is illustrated in Fig. 2

### Feature importance

To understand the features related to DILI, the model prediction was analyzed in the following two ways to recognize the general-to-specific patterns: (i) identifying important features of the overall DILI predictions and (ii) highlighting specific molecular substructures.

1. To identify the general important features of the overall DILI predictions, permutation feature importance was used to identify the feature importance in the RF, LGBM, and LR models. Permutation feature importance is the prominent representative of feature importance measures as it's model-agnostic [41]. The permutation feature importance was implemented in three steps: (1) single feature value in the test dataset was randomly shuffled while keeping the other features unchanged, (2) using the shuffled feature value, new predictions and evaluations were made, and (3) the feature importance was scored based on the performance differences between the original prediction and the new predictions. The larger performance difference obtained a higher score, indicating that the feature contributed significantly to the prediction. Therefore, through the permutation feature importance, the features which had a predictive power overall in each model were detected. In addition, in the LR, the coefficient was used to identify feature importance. As with permutation feature importance, the larger the coefficient, the more influence the feature had on the model.

These methods enabled the identification of feature importance across the entire datasets.

**Fig. 3:**
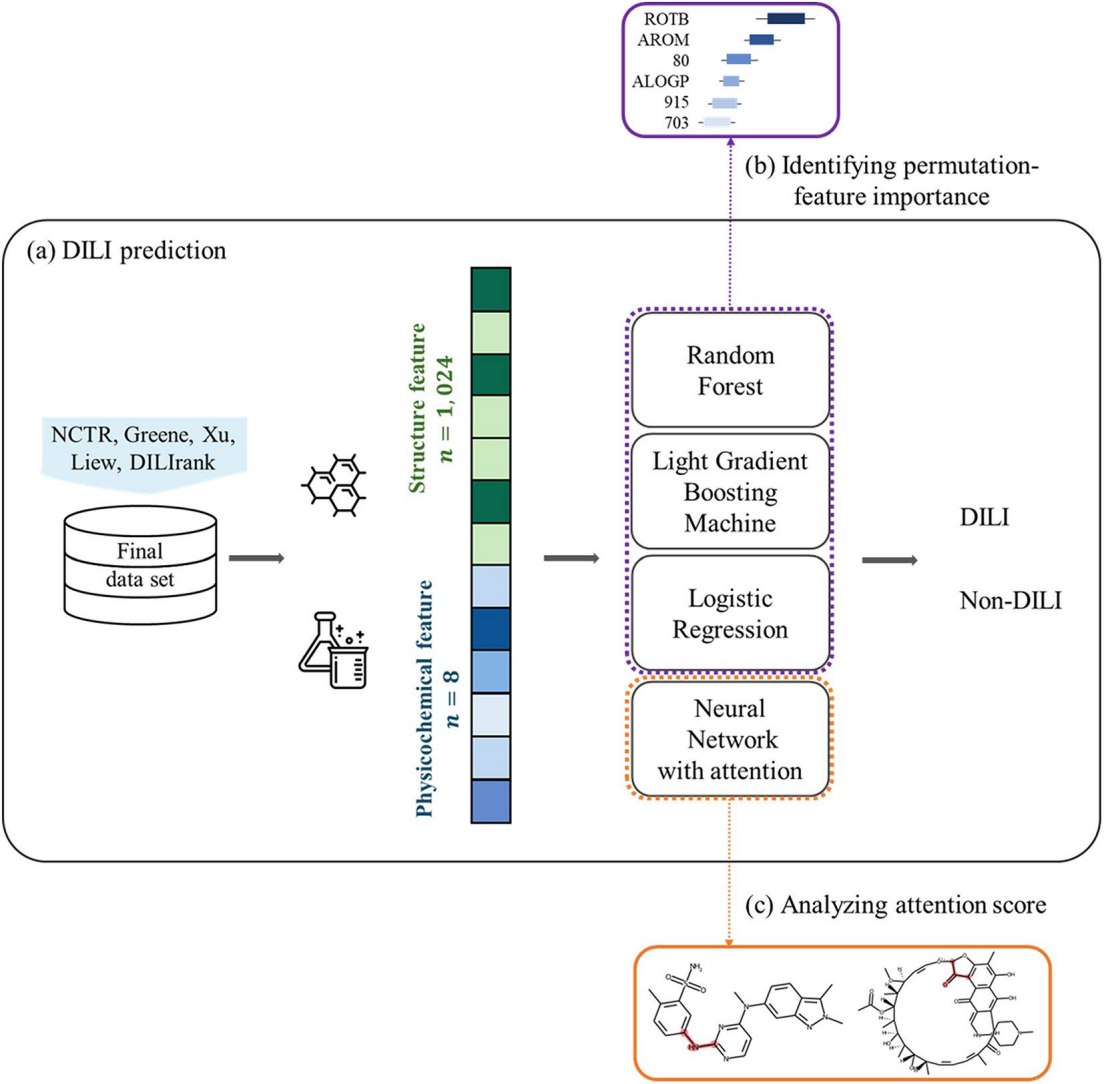
The overall experimental procedure. **a** The structural and physicochemical descriptors of the dataset were calculated. Both features were used as the input, and the DILI label as the output. The RF, LGBM, LR, and NN with attention were used. To interpret the models, **b** identifying important features of the overall DILI predictions using the permutation feature importance and **c** highlighting specific molecular substructures were conducted by analyzing the attention weight

2. Highlighting the specific molecular substructures as mentioned in the ‘Machine Learning Models’ section, the attention weight implicitly indicates the contribution of the substructure of the compound to DILI. With the ECFP6, each bit where the attention weight was calculated corresponded to the molecular substructure. A higher attention weight indicated that the substructure’s corresponding feature was closely associated with toxicity. True positive compounds and their molecular substructures with a high attention weight were analyzed. The experimental procedure is illustrated in Fig. 3

### Model evaluation

We evaluated machine learning model using two methods. First, a hold-out validation was performed with a single test to validate the models. NCTR, Greene, Xu, and Liew datasets was used as training set and DILIrank was used as test set. Second, stratifiedKFold was performed to reduce bias in the data. The dataset was shuffled before being split then distributed based on the proportions of DILI negative and DILI positive compounds. Then, the models were validated by repeated stratified tenfold cross validation.

After each model training, all models were evaluated based on accuracy, sensitivity, specificity, precision, and F1 score, which were calculated as follows. In this study, all models were evaluated based on accuracy, sensitivity, specificity, precision, and F1 score, which were calculated as follows:

$$Accuracy= \frac{TP+TN}{TP+TN+FP+FN} ,$$

$$Sensitivity= \frac{TP}{TP+FN} ,$$

$$Specificity= \frac{TN}{TN+FP} ,$$

$$Precision= \frac{TP}{TP+FP} ,$$

4 $$F1\, score= 2 \times \frac{Pre \times Sen}{Pre+Sen}$$

where TP, FP, TN, and FN represent true positive, false positive, true negative, and false negative, respectively. The area under the receiver operating characteristic (AUROC) and the area under the precision-recall curve (AUPRC) were also utilized to evaluate the prediction performance of the models. An AUROC and AUPRC of 0.5 suggests a random classifier, while an AUROC of 1 represents a perfect classifier.

## Results and discussion

### Performance of machine learning models for DILI prediction

**Fig. 4:**
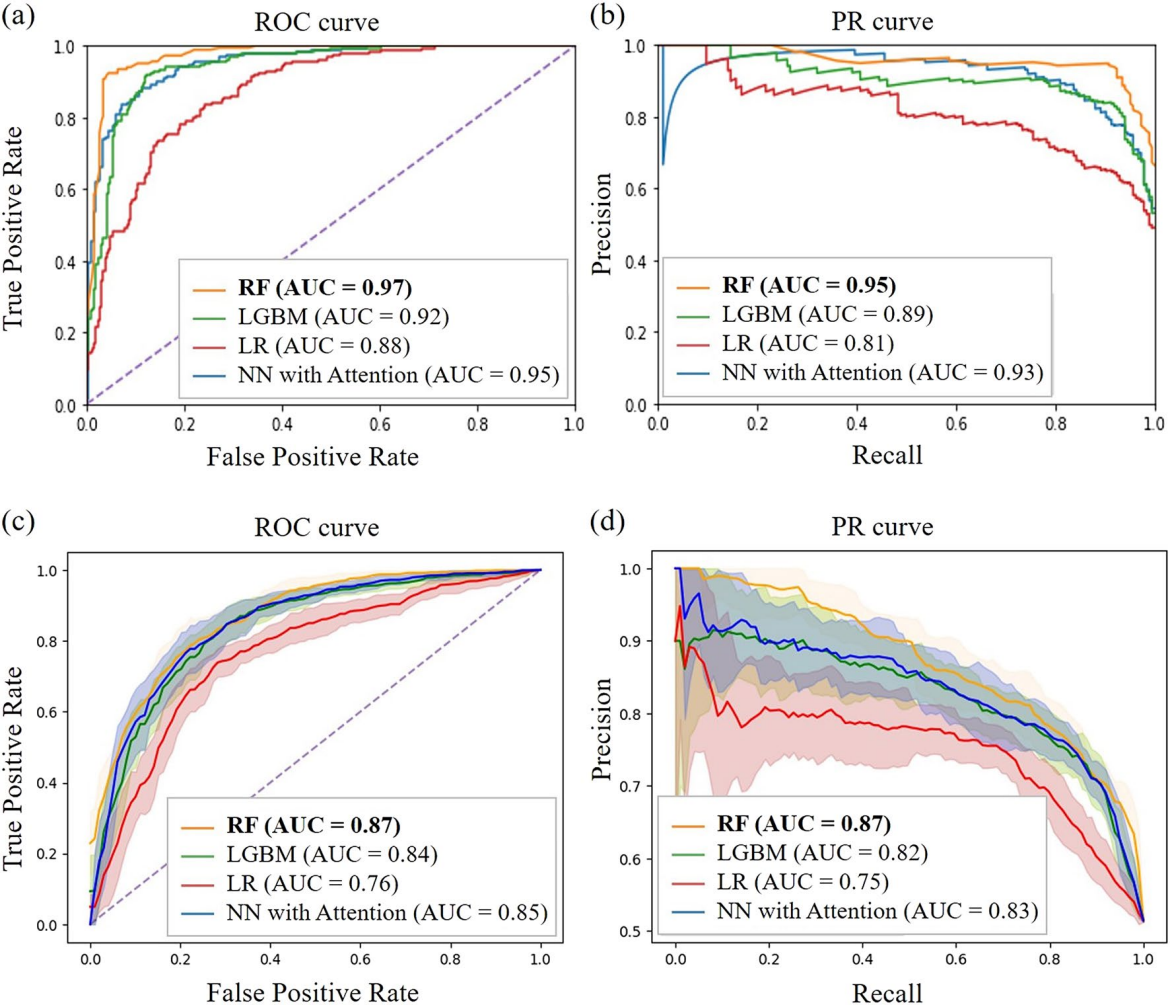
**a** AUROC and **b** AUPR performance of hold-out validation. Four data sets, NCTR, Greene, Xu, and Liew, were used as the training set and the DILIrank was only used as the test set. **c** AUROC and **d** AUPR performance of stratified tenfold cross validation. The shaded region indicates 95% confidence interval

Four machine learning models were evaluated and compared to the performance of a previous study by Nguyen-Vo et al., which generated a model by applying a CNN model based on molecular fingerprint-embedded features [16]. The model by Nguyen-Vo et al. was trained with public datasets and used the DILIrank dataset as an independent set, which was the same test set that was used in this study. The performance of the models with hold-out validation and stratified k-fold cross validation is shown in Additional file 1: Tables S2 and S3. Additionally, the AUROC and AUPR performances are illustrated in Fig. 4

The performance calculated with hold-out validations are illustrated in Fig. 4a, b. In hold-out validation, accuracy varied from 0.81 to 0.90, sensitivity varied from 0.77 to 0.96, specificity varied from 0.62 to 0.90, precision varied from 0.74 to 0.85, and the F1 score varied from 0.77 to 0.89. The AUROC varied from 0.88 to 0.97, and the AUPRC varied from 0.81 to 0.95. The RF model obtained the highest accuracy, sensitivity, F1 score, AUROC, and AUPRC. The NN with attention model provided the highest specificity and precision but gave a relatively low sensitivity. The CNN and the proposed RF models had comparable AUROC performance, with an AUROC of 0.96 and 0.97, respectively. In summary, the RF model was optimal with an accuracy of 0.90, sensitivity of 0.96, specificity of 0.87, precision of 0.83, F1 score of 0.89, AUROC of 0.97, and AUPR of 0.95. The performance calculated with stratified tenfold cross validation are illustrated in Fig. 4c, d. All values are average scores of tenfold. In stratified tenfold cross validation, accuracy varied from 0.72 to 0.78, sensitivity varied from 0.71 to 0.80, specificity varied from 0.72 to 0.76, precision varied from 0.73 to 0.78. The RF model obtained the highest accuracy, sensitivity, specificity, precision, and F1 score. The AUROC varied from 0.76 to 0.87, and the AUPRC varied from 0.75 to 0.87. The RF model obtained the highest accuracy, sensitivity, specificity, precision, F1 score, AUROC, and AUPRC. The NN with attention model provided the highest specificity. In summary, the RF model was optimal with an accuracy of 0.78, sensitivity of 0.80, specificity of 0.76, precision of 0.78, F1 score of 0.79, AUROC of 0.87, and AUPR of 0.0.87.

### Overall feature importance

A total of 1032 features, including structural features (*n* = 1024) and physicochemical descriptors (*n* = 8), were used for model development. Among them, the features which were generally important in predicting DILI were determined. All features were ranked by measuring the effect of the permutation of the variables on performance. To achieve robustness, the permutations were repeated 50 times, and the AUROC was used as the performance metric. The permutation feature importance results obtained from the RF, LGBM, and LR models are illustrated in Fig. 4. Of the 1032 features, the 10 most important features obtained by the model are shown. In the y-axis of the figures, the word indicates one of the physicochemical descriptors, and the number indicates one of the structural features. Additionally, the top 3% most feature importance results are displayed in Additional file 1: Fig. S4.

**Fig. 5:**
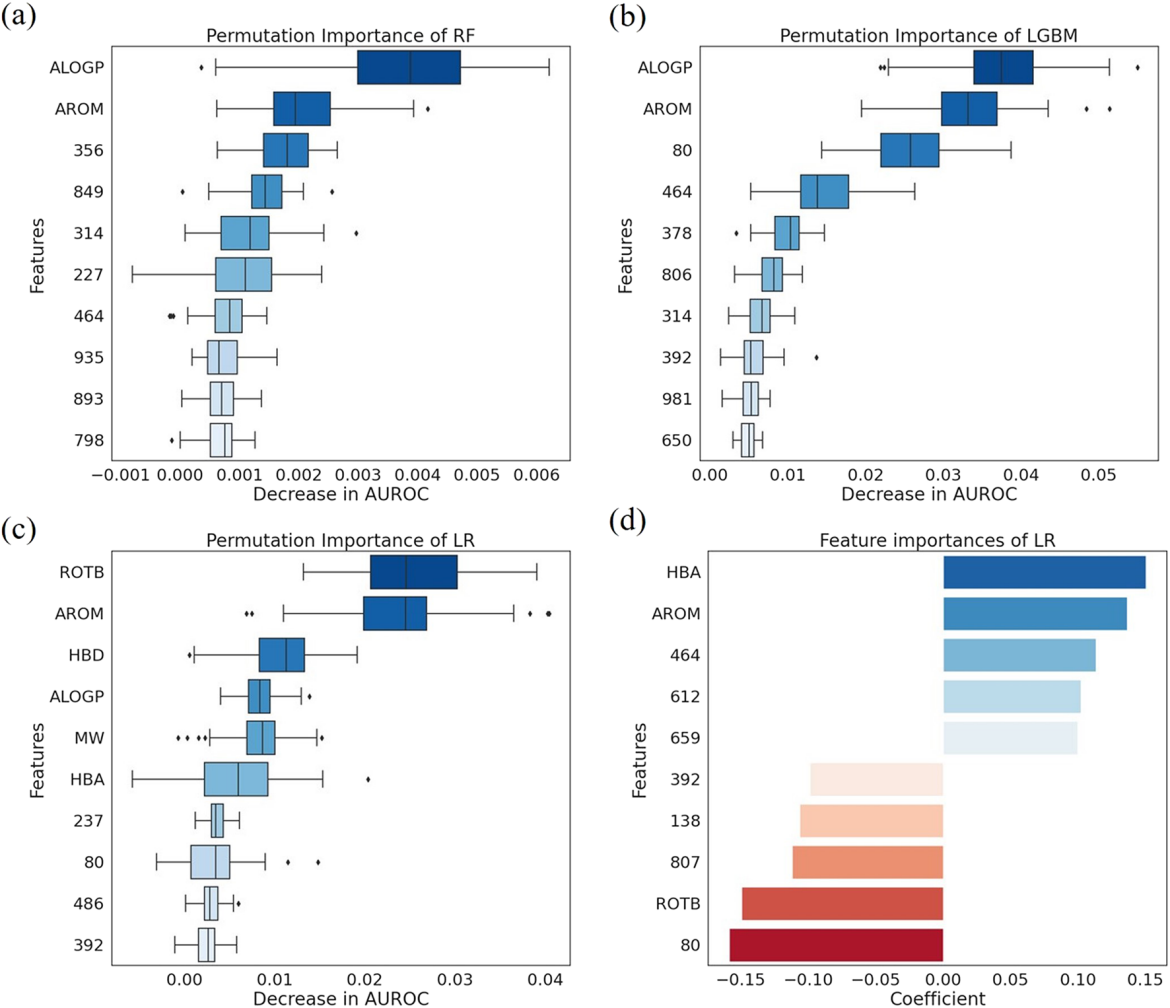
The permutation feature importance of the machine learning models. **a-c** The 10 most important features were ranked, and their boxplots show the distribution of the decrease in the AUROC score. The lower the saturation, the higher the importance score of the feature. **d** The 10 most important features were analyzed with the coefficient of the LR. Features related to a positive DILI prediction are shown in blue, and those related to a negative DILI prediction are shown in red. The lower the saturation, the higher the importance score of the feature. Large absolute means that feature is important

In the RF model, the substructural features of 356, 849, 314, 227, 464, 935, 893, and 798 showed a high importance in the order (Fig. 5a). Additional file 1: Table S4 shows the molecular substructures corresponding to these features. For the physicochemical descriptors, ALOGP and AROM showed high importance in the order. ALOGP, the most important feature in the RF model, was significantly higher than other features, with a mean of 0.006 and a standard deviation (*SD*) of 0.0029 for the decrease in the AUROC. In the LGBM model, the substructural features of 80, 464, 378, 806, 314, 392, 981, and 650 showed a high importance in the order (Fig. 5b). Additional file 1: Table S5 shows the molecular substructures corresponding to these features. For the physicochemical descriptors, ALOGP and AROM showed high importance in the order. ALOGP, the most important feature in the LGBM model, was significantly higher than other features, with a mean of 0.0521 and an *SD* of 0.016 for the decrease in the AUROC. In the LR model, substructural features of 237, 80, 486, and 392 showed a high importance in the order (Fig. 5c). Additional file 1: Table S6 shows the molecular substructures corresponding to these features. For the physicochemical descriptors, ROTB, AROM, HBD, ALOGP, MW, and HBA showed high importance in the order. ROTB was the most important feature in the LR model, with a mean of 0.0375 and an *SD* of 0.0185. The feature importance with coefficient in the LR model was further analyzed (Fig. 5d). Substructural features of 464, 612, 659, 392, 138, 807, and 80 showed a high importance in the order. Additional file 1: Table S7 shows the molecular substructures corresponding to these features. For the physicochemical descriptors, HBA, AROM, and ROTB showed high importance in the order. The feature importance in the LR model was analyzed in two ways. ROTB, AROM, HBA, and substructural features of 80 and 392 were found to be of common importance in both methods.

In all models, the importance of each feature was identified. Analyzing the results of the feature importance, substructural features of 80 and 464 showed a high correlation with the DILI prediction. As mentioned in the ‘Molecular descriptor’ section, binary vectors of the ECFP6 represent the molecular substructures in bits through a hash function. In this process, there may be collisions between bits where the features and molecular structures do not correspond exactly one-to-one. Thus, the molecular substructures corresponding to the features of 464 and 80 were additionally identified. The molecular substructures associated with the features of 464 and 80 were identified in several compounds and are shown in Table 2.

**Table 2:** Molecular substructures corresponding to features

Feature	Substructure	Description	SMILES
80		2,3-Dimethylindazole	Cc1c2ccccc2nn1C
		(1S,2S)-1-cyclohexyl-2-(methylamino)propan-1-ol	ccc(cc)[C@H](O)[C@H](C)NC
		2-isopropoxypropane	C[C@H](C)Oc(c)c
		**Acetohydrazide**	**cC(= O)NN**
		Pentan-3-ol	CCC(O)CC
464		2,3-Dichloro-4-methylanisole	COc1ccc(C)c(Cl)c1Cl
		4-Ethyl-3-methylene-1,2,3,4-tetrahydroquinoline	cc1cNc2ccccc2C1CC
		Ethenamine	ccn
		**Methylhydrazine**	**CNN**
		1,2-dimethoxy-4-methylbenzene	cc1ccc(OC)c(OC)c1
		2-ethyl-3,5-dimethylbenzene-1,4-diol	CCc1c(O)cc(C)c(O)c1C
		(Z)-3-choloro-4-fluoropenta-1,3-diene	ccc(Cl)c(c)F
		1-chloro-3-methylbenzene	Cc1cccc(Cl)c1
		2,3-dichloro-1-methoxy-4-methyldbenzene	COc1ccc(C)c(Cl)c1Cl
		1-(4-chloro-2-methylphenyl)pyrrolidine	cn(c)-c1ccc(Cl)cc1C
		Trimethoxy(methyl)silane	CO[Si](C)(OC)OC
		3-hydroxy-2-(1-hydroxypropyl)pentanoic acid	CCC(O)C(C(= O)O)C(O)CC

The *dashed lines* represents conjugated double bonds and the *bold font* are molecular substructures known to be related to DILI

There are several studies that support this analysis. The substructural feature 80 corresponds to several molecular substructures. Among them, Acetohydrazide exacerbates liver cell injuries [42]. Hydrozine, one of the molecular substructures corresponding to the substructural feature 464, is known to cause liver damage [42, 43]. The pharmaceutical industry paid attention to controlling hydrazine levels due to liver toxicity. Of the physicochemical descriptors, AROM had important overall importance in the entire model. Next, ALOGP showed a high correlation with DILI in three feature importances. Particularly, ALOGP was the highest predictor of DILI in the RF and LGBM models. In the permutation feature importance of the LR model, the physicochemical descriptorshad a significant impact on predicting DILI, and six of the ten most important features were, in order, ROTB, AROM, HBD, ALOGP, MW, and HBA. ALOGP and AROM are known to be associated with the risk of DILI [26, 44–46]. ALOGP is used to measure a drug’s lipophilicity, and AROM is the number of aromatic rings. DILI-positive drugs had higher lipophilic and greater aromatic ring counts than DILI-negative drugs [46]. This is consistent with these findings, which confirmed that AROM has a positive correlation with DILI.

### Importance of specific molecular substructures

To investigate which parts of the molecule played an important role in predicting DILI, several compounds in the test data were analyzed. Among the true positive compounds, the compounds with high prediction probabilities (p $\ge$ 0.8) were Pazopanib, Rifampin, Itraconazole, Imatinib, Dactinomycin, Tasosartan, and Atorvastatin. The molecular substructures that significantly contributed to the DILI were highlighted through their attention weights. The three most important molecular substructures with high attention weights in each compound are displayed in Additional file 1: Fig. S5. Several highlighted results of the analysis were further compared with external literature.

**Fig. 6:**
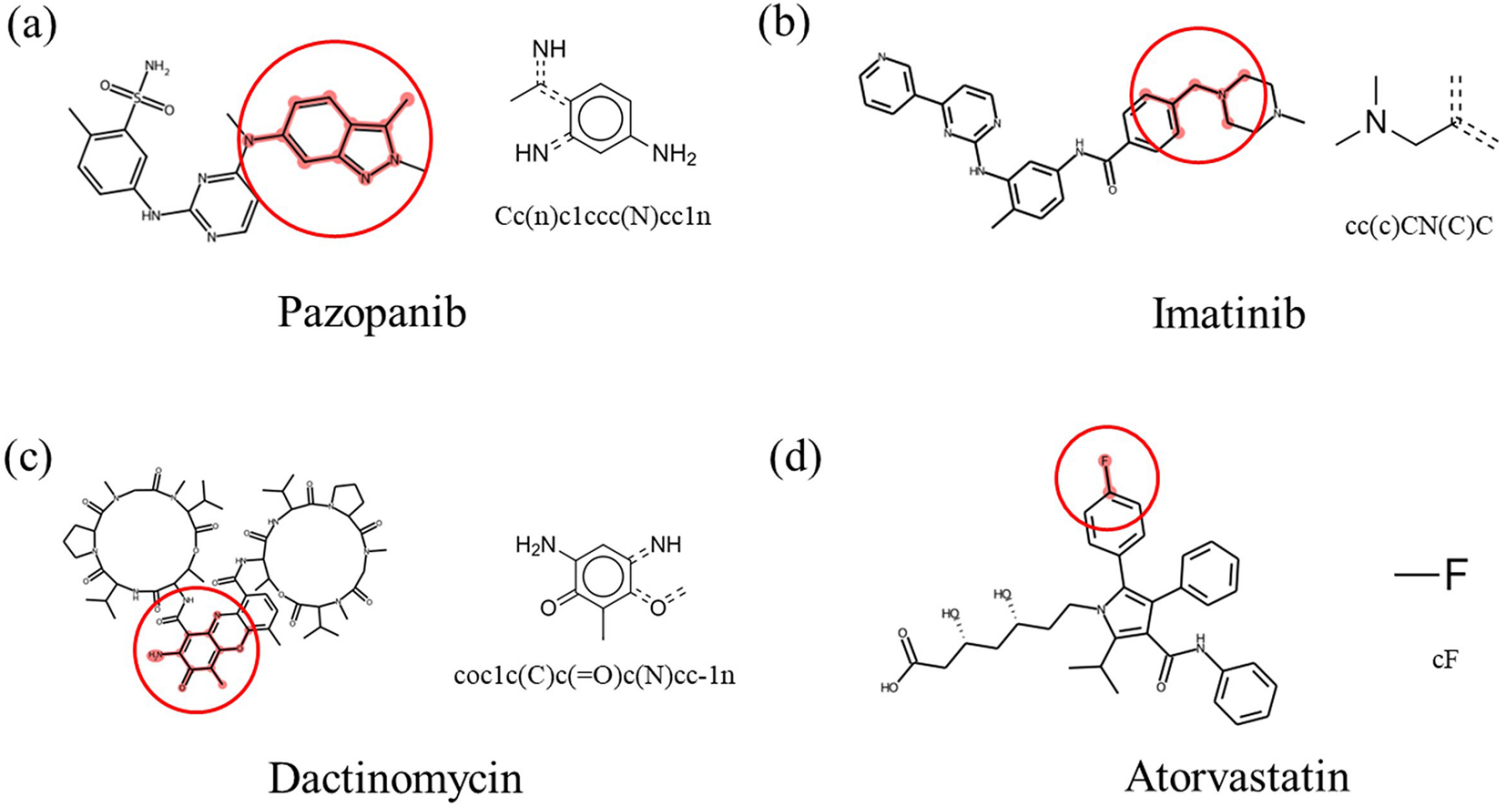
Highlighted molecular substructures in **a** Pazopanib, **b** Imatinib, **c** Dactinomycin, and **d** Atorvastatin. Molecular substructures that contributed to the DILI predictions are highlighted (left), and the molecular substructures and their SMILES are presented (right). All four molecular substructures are known to be responsible for DILI. The *dashed lines* indicate conjugated double bonds

Aniline derivatives commonly in Pazopanib and Dactinomycin influenced the prediction of DILI (Fig. 6a, c). Many compounds with aniline moieties are known to be mutagenic, and structure alerts are frequently marked in substituents known to form anilines [47, 48]. In particular, the aniline derivatives in Pazopanib are identified as a structural alert in the analysis of drugs associated with black box warnings due to hepatotoxicity [47]. One of the molecular substructures that significantly contributed to Imatinib’s DILI positive prediction was the trimethylamine group (Fig. 6b). The other DILI prediction models found that trimethylamine groups only appeared in DILI positives [49]. Finally, it was found that fluorine bonded to a sp3 carbon contributed the most to the DILI prediction of Atorvastatin (Fig. 6d). In the presence of fluorine atoms, drug lipophilicity increases, which could increase the intracellular concentration of hepatotoxic drugs [12, 50, 51]. This study confirmed that this model can determine well-known DILI structural alerts. Therefore, the highlighted molecular substructures have not yet been reported but may potentially affect DILI.

### Limitations of the study

This study can be further improved by considering the following. The Morgan fingerprint algorithm can affect the model prediction. The ECFP6 was used to capture molecular substructures with radius of 3. Therefore, molecular substructures with larger radius sizes might have been missed. In addition, a few molecular substructures were collided with one feature due to the hash function used to calculate the structure features. Further research is needed to resolve this feature ambiguity. Lastly, this study did not consider dose-dependent DILI. The dose is an important feature because the induction of DILI may vary with the dose. However, this study lacked detailed data sources and standard doses of the drugs. When all the limitations are addressed, the DILI prediction will be more accurate.

## Conclusion

An interpretable prediction for DILI was proposed in this study. This study applied machine learning models using molecular substructural and physicochemical descriptors, and the models achieved overall AUROC values ranging from 0.88 to 0.97. These models tried to interpret the DILI predictions in two ways. First, through permutation feature importance, the molecular substructure features of 80 and 464 and the physicochemical descriptors of AROM and ALOGP were identified to be highly important. These features were previously reported to have significant correlations with DILI. However, during the process of converting molecular structures into bits, one feature may be assigned to multiple molecular substructures. Therefore, an analysis of the attention weight for each compound was further conducted to identify which molecular substructure had a substantial impact on the DILI prediction. This analysis showed that specific molecular substructures, such as aniline derivatives, trimethylamine groups, and fluorocarbons, significantly contribute to the DILI prediction. These substructures are well-known structural alerts highly associated with DILI, confirming that the model performed well in predicting DILI. The proposed model enables proactive DILI prediction for compounds during drug development, thereby enhancing drug safety and preventing potential side effects in advance. Additionally, the model's interpretability is expected to aid researchers in making modifications or alternative exploration of those substructures to mitigate DILI, as it can identify risks associated with specific molecular substructures.

### Supplementary Information

**Additional file 1: Figure S1.** Mean and standard deviation (Std) for values of 0 and 1 of molecular substructure features. **Figure S2.** Distribution of MW, ALOGP, HBA, HBD, PSA, ROTB, AROM, and ALERTS. **Figure S3.** Distribution of MW, ALOGP, HBA, HBD, PSA, ROTB, AROM, and ALERTS after applied standard scaler (mean = 0, variance = 1). **Figure S4.** The permutation feature importance of the machine learning models. **a–c** The top 3% important features were ranked, and their boxplots show the distribution of the decrease in the AUROC score. The lower the saturation, the higher the importance score of the feature. **d** The top 3% most important features were analyzed with the coefficient of the LR. Features related to a positive DILI prediction are shown in blue, and those related to a negative DILI prediction are shown in red. The lower the saturation, the higher the importance score of the feature. Large absolute means that feature is important. **Figure S5.** The three most important molecular substructures in **a** Pazopanib, **b** Rifampin, **c** Itraconazole, **d** Imatinib, **e** Dactinomycin, and **f** Tasosartan. The highlights in red were features that contributed significantly to the DILI prediction. An attention weight is presented under each molecular substructure, and they are arranged in order of highest to lowest attention weights. **Table S1.** Hyperparameter search details and optimal values for the machine learning models. **Table S2.** Performance of the RF, LGBM, LR, NN with attention, and CNN models in hold-out validation. **Table S3.** Performance of the RF, LGBM, LR, NN with attention, and CNN models in stratified k-fold cross validation. All values are average scores of 10 fold. **Table S4.** Molecular substructures corresponding to features with high importance in the RF model. **Table S5.** Molecular substructures corresponding to features with high importance in the LGBM model. **Table S6.** Molecular substructures corresponding to features with high importance in the LR model using permutation feature importance. **Table S7**. Molecular substructures corresponding to features with high importance in the LR model.

## Data Availability

The data sets used in this study, as well as the essential code, are available on GitHub at https://github.com/bmil-jnu/InterDILI.
