# Supplementary material for: InterDILI: interpretable prediction of drug-induced liver injury through permutation feature importance and attention mechanism
Source: J Cheminform. 2024 Jan 3;16:1. doi: 10.1186/s13321-023-00796-8 (PMC10765872; doi:10.1186/s13321-023-00796-8)
Supplement: Supplementary file 1 — Additional file 1: Figure S1. Mean and standard deviation (Std) for values of 0 and 1 of molecular substructure features. Figure S2. Distribution of MW, ALOGP, HBA, HBD, PSA, ROTB, AROM, and ALERTS. Figure S3. Distribution of MW, ALOGP, HBA, HBD, PSA, ROTB, AROM, and ALERTS after applied standard scaler (mean = 0, variance = 1). Figure S4. The permutation feature importance of the machine learning models. a–c The top 3% important features were ranked, and their boxplots show the distribution of the decrease in the AUROC score. The lower the saturation, the higher the importance score of the feature. d The top 3% most important features were analyzed with the coefficient of the LR. Features related to a positive DILI prediction are shown in blue, and those related to a negative DILI prediction are shown in red. The lower the saturation, the higher the importance score of the feature. Large absolute means that feature is important. Figure S5. The three most important molecular substructures in a Pazopanib, b Rifampin, c Itraconazole, d Imatinib, e Dactinomycin, and f Tasosartan. The highlights in red were features that contributed significantly to the DILI prediction. An attention weight is presented under each molecular substructure, and they are arranged in order of highest to lowest attention weights. Table S1. Hyperparameter search details and optimal values for the machine learning models. Table S2. Performance of the RF, LGBM, LR, NN with attention, and CNN models in hold-out validation. Table S3. Performance of the RF, LGBM, LR, NN with attention, and CNN models in stratified k-fold cross validation. All values are average scores of 10 fold. Table S4. Molecular substructures corresponding to features with high importance in the RF model. Table S5. Molecular substructures corresponding to features with high importance in the LGBM model. Table S6. Molecular substructures corresponding to features with high importance in the LR model using permutation feature impo [file 13321_2023_796_MOESM1_ESM.docx]

# **InterDILI: interpretable prediction of drug-induced liver injury through permutation feature importance and attention mechanism**

Soyeon Lee^1,2^, and Sunyong Yoo^1 *^

^1^Department of ICT Convergence System Engineering, Chonnam National University, Gwangju 61186, Republic of Korea

^2^Division of Bioresources Bank, Honam National Institute of Biological Resources, Mokpo 58762, Republic of Korea

# **Additional Figure**


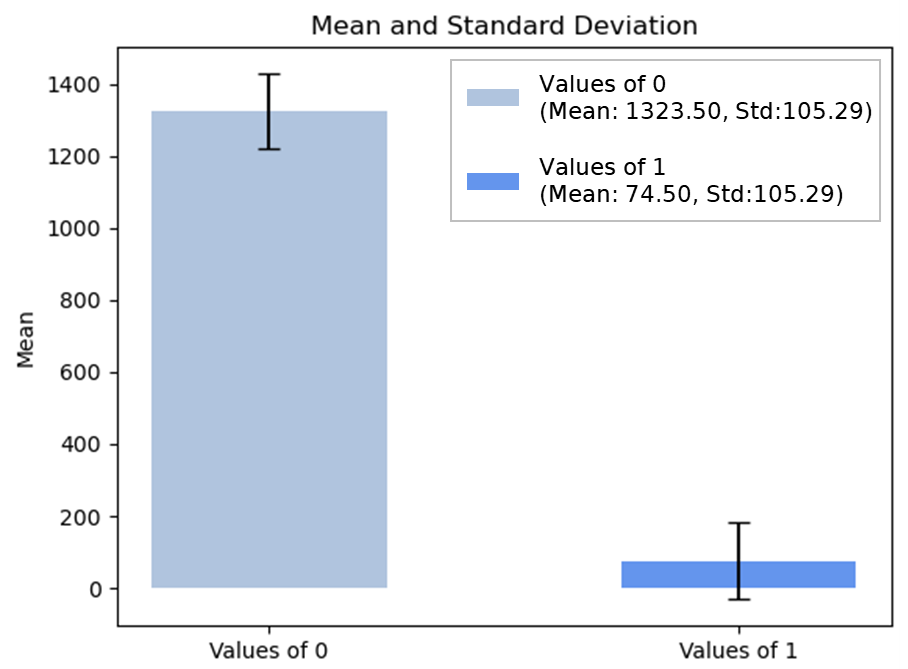


**Figure S1** Mean and standard deviation (Std) for values of 0 and 1 of molecular substructure features.


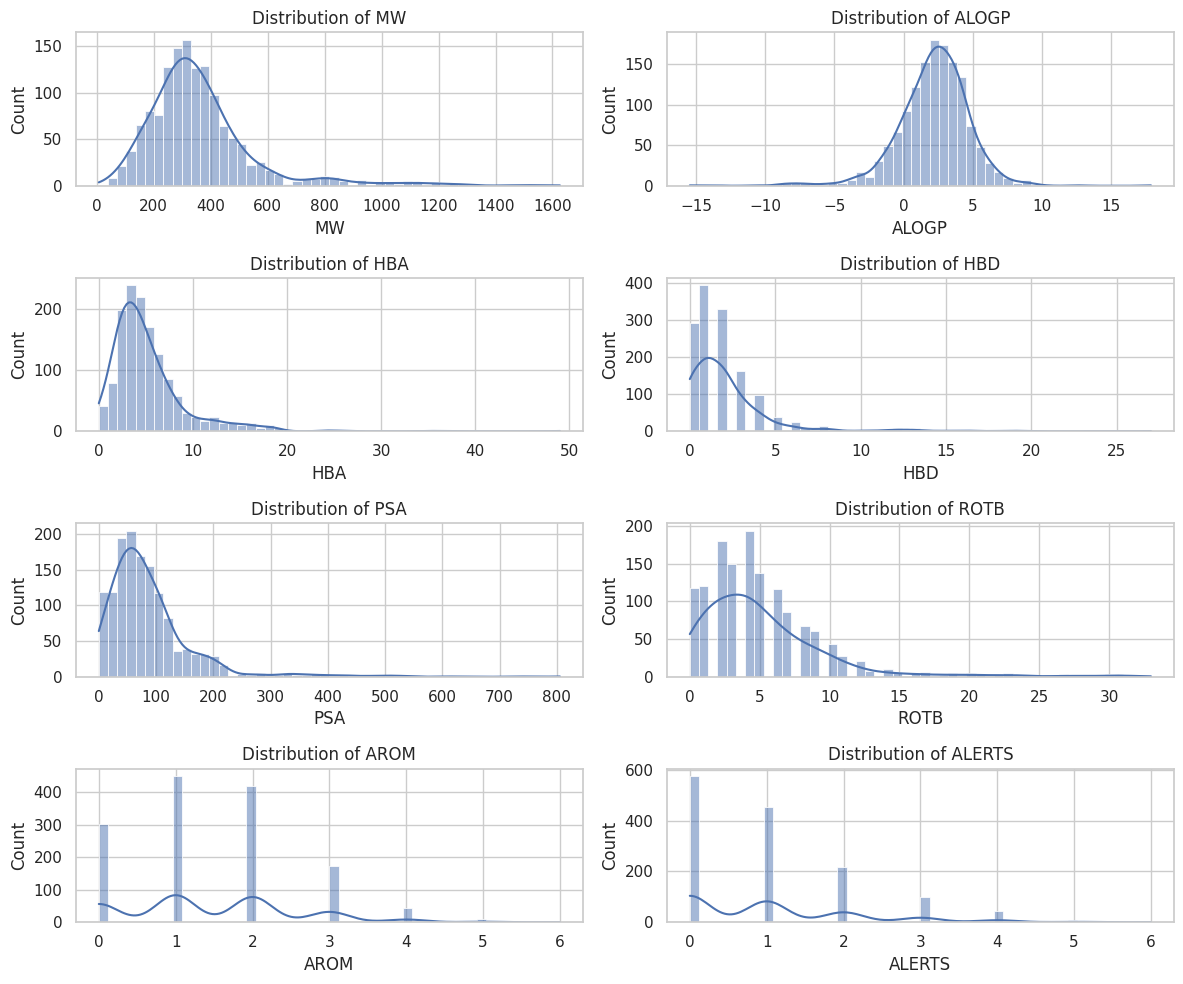


**Figure S2** Distribution of MW, ALOGP, HBA, HBD, PSA, ROTB, AROM, and ALERTS.


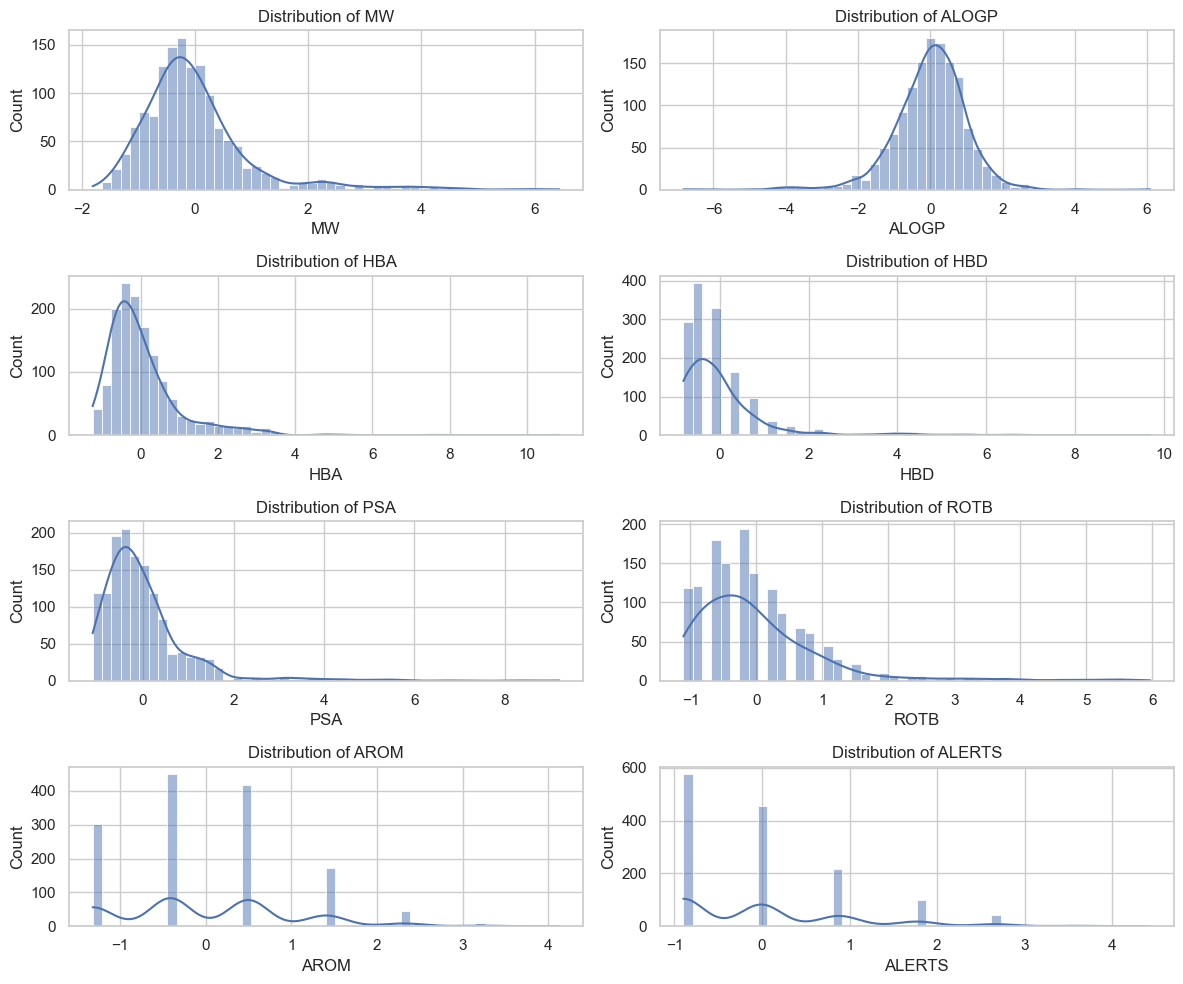


**Figure S3** Distribution of MW, ALOGP, HBA, HBD, PSA, ROTB, AROM, and ALERTS after applied standard scaler (mean = 0, variance = 1).

**
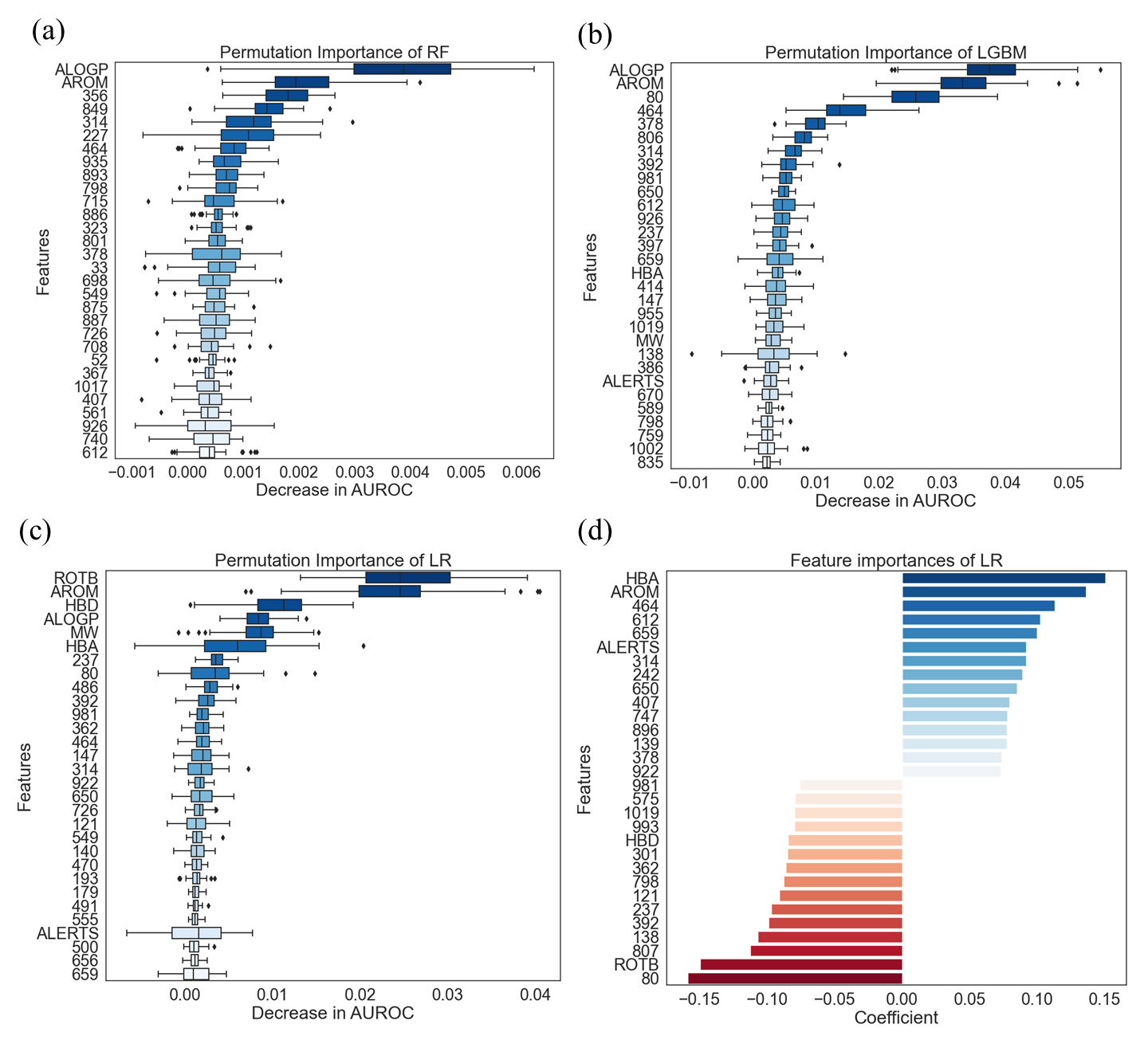
**

**Figure S4** The permutation feature importance of the machine learning models. (**a-c**) The top 3% important features were ranked, and their boxplots show the distribution of the decrease in the AUROC score. The lower the saturation, the higher the importance score of the feature. (**d**) The top 3% most important features were analyzed with the coefficient of the LR. Features related to a positive DILI prediction are shown in blue, and those related to a negative DILI prediction are shown in red. The lower the saturation, the higher the importance score of the feature. Large absolute means that feature is important.

**
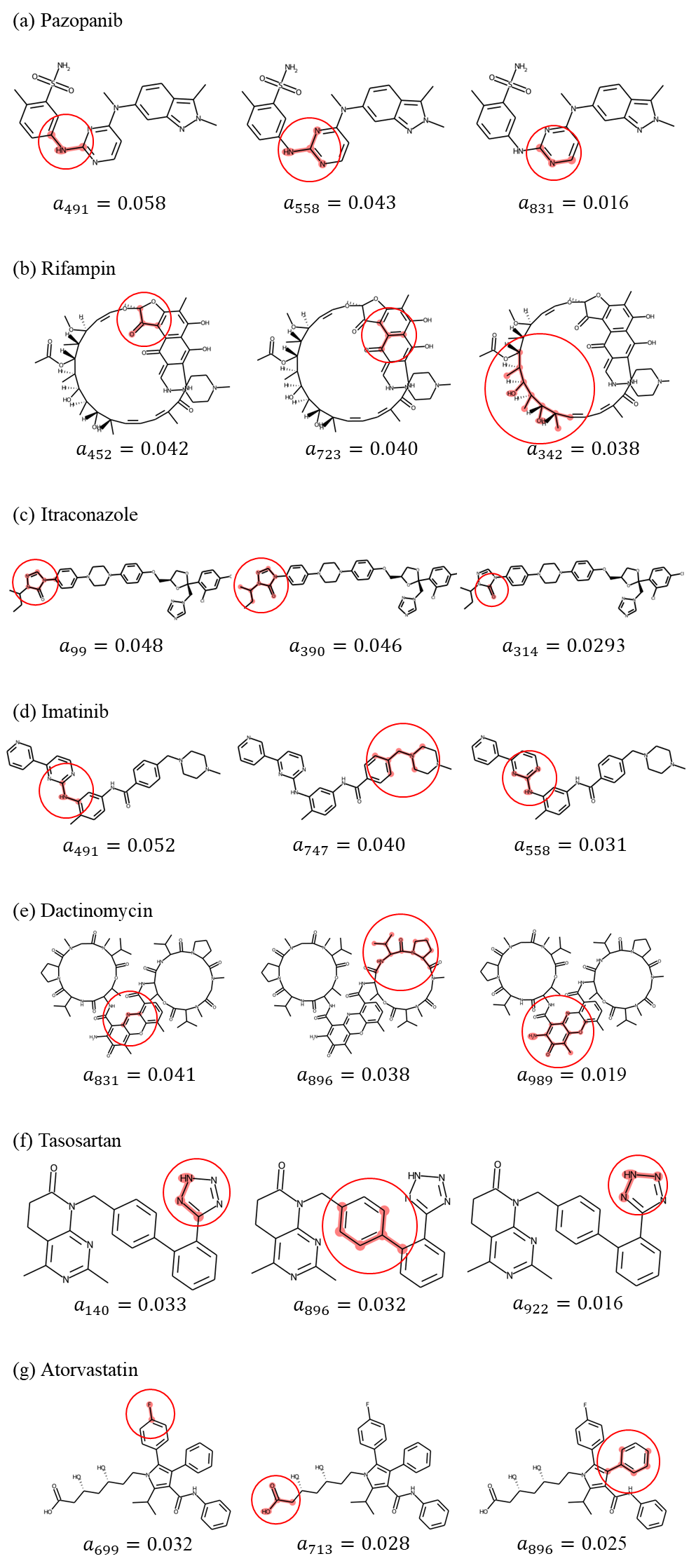
**

**Figure S5** The three most important molecular substructures in (a) Pazopanib, (b) Rifampin, (c) Itraconazole, (d) Imatinib, (e) Dactinomycin, and (f) Tasosartan. The highlights in red were features that contributed significantly to the DILI prediction. An attention weight is presented under each molecular substructure, and they are arranged in order of highest to lowest attention weights.

**Additional Table**

**Table S1** Hyperparameter search details and optimal values for the machine learning models.

| **Model** | **Parameters** | **Ranges** | **Optimal values** |
| --- | --- | --- | --- |
| RF | Number of trees | [10, 30, 50, 70, 90] | 90 |
|  | Max number of features | [18, 20, 22, 24] | 18 |
|  | Max depth | [100, 130, 150, 170] | 130 |
| LGBM | Max number of leaves | [20, 40, 60, 80, 100] | 60 |
|  | Number of boosting iterations | [10, 30, 50, 70, 90] | 30 |
|  | Minimal number of data | [5, 10, 15] | 10 |
|  | Max depth | [3, 5, 10] | 10 |
| LR | Maximum number of iterations | [1000, 3000, 5000, 7000, 10000] | 1,000 |
|  | Strength of the regularization | [0.001, 0.01, 0.1, 1, 10, 100] | 0.01 |
| NN with attention | Number of layers | [1, 2, 3] | 1 |
|  | Number of nodes | [16, 32, 64, 128, 256, 512] | 512 |

**Table S2** Performance of the RF, LGBM, LR, NN with attention, and CNN models in hold-out validation.

| **Model** | **Accuracy** | **Sensitivity** | **Specificity** | **Precision** | **F1 score** |
| --- | --- | --- | --- | --- | --- |
| RF | 0.90 | 0.96 | 0.87 | 0.83 | 0.89 |
| LGBM | 0.84 | 0.91 | 0.73 | 0.74 | 0.82 |
| LR | 0.81 | 0.77 | 0.62 | 0.76 | 0.77 |
| NN with attention | 0.89 | 0.88 | 0.90 | 0.85 | 0.86 |
| CNN [16] | 0.89 | - | - | - | - |

**Table S3** Performance of the RF, LGBM, LR, NN with attention, and CNN models in stratified k-fold cross validation. All values are average scores of 10 fold.

| **Model** | **Accuracy** | **Sensitivity** | **Specificity** | **Precision** | **F1 score** |
| --- | --- | --- | --- | --- | --- |
| RF | **0.78** | **0.80** | **0.76** | **0.78** | **0.79** |
| LGBM | 0.77 | 0.78 | 0.75 | 0.77 | 0.77 |
| LR | 0.72 | 0.71 | 0.72 | 0.73 | 0.72 |
| NN with attention | 0.75 | 0.74 | **0.76** | 0.77 | 0.74 |

**Table S4** Molecular substructures corresponding to features with high importance in the RF model.

| **Feature** | **Substructure** | **SMILES** |
| --- | --- | --- |
| 356 |  | C[C@H](C)O |
| 849 |  | cNC(C)=O |
|  |  | C[C@@H](C)O |
|  |  | C[C@H](C)O |
| 314 |  | C=O, c=O |
|  |  | CCN(CC)CC(=O)O |
| 227 |  | CO |
|  |  | CC[C@@H](NC)B(O)O |
|  |  | CC(C)O[C@@H]1O[C@@H](C)C[C@H](O)[C@H]1O |
|  |  | C=Cc |
| 935 |  | cnc(=S)c(c)[nH] |
|  |  | CCC[C@@H](C)N |
|  |  | CCCC(C)N |
|  |  | cc(=O)nc([nH])N |
|  |  | CCC[C@H](C)N |
|  |  | C[C@](C)(O)[C@@H](Cc)[N+](C)(C)C |
| 893 |  | C=O |
|  |  | CNC(=O)C(C)N |
|  |  | CCCCN |
|  |  | COCCO |
|  |  | CCCn1nc(C)n(C)c1=O |
|  |  | CCC(CC)NC |
|  |  | cc(C)oc(c)C |
|  |  | CCc1cnc[nH]1 |
| 798 |  | CC(N)=O |
|  |  | CCCC[NH2+] |
|  |  | cc1cccc(OC)c1 |
|  |  | C[C@@H](C)O |
|  |  | COc1ccccc1O |
|  |  | ccc(cc)CC |
|  |  | CC(=O)NC(C)C |
|  |  | C[C@H](C)O |
|  |  | CC[C@](O)(C(=O)CO)C(C)(C)C |
|  |  | CCC(OC)C(N)=O |
|  |  | cc(c)-c1ccncn1n |
|  |  | ccc(CC)c(c)C |
|  |  | CCC(CC)([C@H](C)C)C(C)(C)O |
|  |  | C[C@@H](N)CC(N)=O |
|  |  | CCCCN |
|  |  | C[C@@H]1CC=CCN1CC |
|  |  | CN[C@@H](CC(C)C)C(=O)O |
|  |  | CC(N)CC(N)=O |
|  |  | cc(c)C[C@H](NS(C)(=O)=O)C(=O)O |

**Table S5** Molecular substructures corresponding to features with high importance in the LGBM model.

| **Feature** | **Substructure** | **SMILES** |
| --- | --- | --- |
| 378 |  | ccc(cc)Oc(cc)cc |
|  |  | cc(c)C1(CC)C(=O)NC(=O)NC1=O |
|  |  | C=C(C)NC(=O)/C(C)=C\C |
|  |  | cc(c)C(=O)c1cc(I)cc(I)c1 |
|  |  | CC[N+] |
|  |  | cc(c)[C@@H](Cc)[N+](C)(C)C |
|  |  | CCNC(C)c |
|  |  | cc(c)[C@@H](O)CN |
|  |  | cc(c)C(Cc)[N+](C)(C)C |
| 806 |  | c[nH]c |
|  |  | Cc1ccccc1N(c)C |
|  |  | C[C@H](N)[C@H](O)C1CCCCC1 |
|  |  | cc(C)cc(c)C |
|  |  | CC(C)(c)[C@@H](Oc)C(C)=O |
|  |  | CCN(C(C)=S)c1ccccc1C(c)=N |
|  |  | CN[C@H](C(=O)NC(C)C)C(C)C |
| 314 |  | C=O, c=O |
|  |  | CCN(CC)CC(=O)O |
| 392 |  | CC |
|  |  | C=C(c)c1ccccc1CCc |
|  |  | cc(c)S |
|  |  | CN/C(NN)=C1\C=CC=CC1=O |
|  |  | CNc, cNC |
|  |  | CN(C)[C@@H](Cc(cc)cc)C(N)=O |
|  |  | CC(c)(c)O |
|  |  | ccc(cc)-c1ccnc(c)n1nc |
|  |  | Cc1cccc2c1CCO2 |
|  |  | Cc(c)cc(-c)n |
|  |  | CC(=O)NCC(=O)N(C)C |
|  |  | C[C@H](N)C(=O)N(C)C |
|  |  | CC(N)C(=O)N(C)C |
|  |  | C=C/C=C/CCC |
| 981 |  | CC(C)C(O)C(C)N |
|  |  | cc1nc(c)c(c(c)C)oc-1c(c)C |
|  |  | Cc1ccsc1C |
|  |  | CN |
|  |  | CC(C)C |
|  |  | cc(c)C(c1ccccc1)C(Cl)ClS |
|  |  | cc(c)I |
| 650 |  | Cc1cccc(-c)c1 |

**Table S6** Molecular substructures corresponding to features with high importance in the LR model using permutation feature importance.

| **Feature** | **Substructure** | **SMILES** |
| --- | --- | --- |
| 237 |  | cc(c)C |
|  |  | CCCN(C)C |
|  |  | cc1c(n)ncn1C |
|  |  | CC[C@@H](CO)NC(C)=O |
|  |  | cc(c)C(CC)c1ccccn1 |
|  |  | cc(c)[C@H](CC)c1ccccn1 |
|  |  | cc(N)nc(n)NS |
|  |  | NCS |
|  |  | c-1ccc2c(C)cccc2n1 |
|  |  | cN(C)C(N(C)C)C(C)(C)c |
|  |  | COCC(C)(C)N |
|  |  | cc(N)nc(n)-n |
|  |  | CNCC(=O)NC(C)C |
|  |  | CCCC(=O)OCC |
|  |  | C=CC(CC(O)C(C)C)OC |
| 486 |  | cC(N)=O |
|  |  | CCOC |
|  |  | O=P |
|  |  | ccc(Cl)cn |
|  |  | cNc1ccc(C)cc1CC(=O)O |
|  |  | cC(O)c1ccccc1 |
|  |  | CN(C)C |
|  |  | CC(C)O |
|  |  | c[nH]c1ccccc1c |
|  |  | CNC(=O)C(C)C |
|  |  | ccc(O)c(c)C |
|  |  | CC(C)[C@@H]1CC[C@H](C)C1(C)C |
|  |  | C=C1N(CC)c2cccc(c)c2C1(C)C |
|  |  | NC(=O)[C@@H](N)CS |
|  |  | NC(=O)C(N)CS |
|  |  | CCN[C@H](C)/C(C)=N/O |
| 392 |  | CC |
|  |  | C=C(c)c1ccccc1CCc |
|  |  | cc(c)S |
|  |  | CN/C(NN)=C1\C=CC=CC1=O |
|  |  | CNc, cNC |
|  |  | CN(C)[C@@H](Cc(cc)cc)C(N)=O |
|  |  | CC(c)(c)O |
|  |  | ccc(cc)-c1ccnc(c)n1nc |
|  |  | Cc1cccc2c1CCO2 |
|  |  | Cc(c)cc(-c)n |
|  |  | CC(=O)NCC(=O)N(C)C |
|  |  | C[C@H](N)C(=O)N(C)C |
|  |  | CC(N)C(=O)N(C)C |
|  |  | C=C/C=C/CCC |

**Table S7** Molecular substructures corresponding to features with high importance in the LR model.

| **Feature** | **Substructure** | **SMILES** |
| --- | --- | --- |
| 464 |  | cc1ccc(OC)c(OC)c1 |
|  |  | CCc1c(O)cc(C)c(O)c1C |
|  |  | ccn |
|  |  | ccc(Cl)c(c)F |
|  |  | Cc1cccc(Cl)c1 |
|  |  | COc1ccc(C)c(Cl)c1Cl |
|  |  | cc1cNc2ccccc2C1CC |
|  |  | CNN |
|  |  | cn(c)-c1ccc(Cl)cc1C |
|  |  | CO[Si](C)(OC)OC |
|  |  | CCC(O)C(C(=O)O)C(O)CC |
| 612 |  | ccc(c(c)=O)c(n)n |
|  |  | CC(N)CC(C)O |
|  |  | CC(C)(C)O |
|  |  | CN(C)CCCl |
|  |  | c[C@H]([N+])Cc1cccc(O)c1 |
|  |  | cC([N+])Cc1cccc(O)c1 |
|  |  | C/C=C/C(C)O |
| 659 |  | C=C(O)NCc |
|  |  | ccc(cc)CN |
|  |  | CN[C@H]1C=C(CO)[C@H](O)C[C@H]1O |
|  |  | cc([nH])C(CC)(CC)OC |
|  |  | ccc(c(c)O)C(C)C |
|  |  | CN(C)CCCCn |
|  |  | ccc(cc)Cn |
|  |  | N[C@@H]1C[C@H](N)[C@@H](O)C[C@H]1O |
|  |  | CCc1ncc[nH]1 |
|  |  | NC1CC(N)C(O)CC1O |
|  |  | CO[C@@H]1[C@@H](N=C(N)N)[C@H](O)[C@@H](N)C[C@H]1O |
|  |  | NCC(=O)N[C@@H](CS)C(N)=O |
|  |  | cc(c)CNC |
|  |  | C=C[C@H]([C@H](C)C)[C@@H](C)O |
| 392 |  | CC |
|  |  | C=C(c)c1ccccc1CCc |
|  |  | cc(c)S |
|  |  | CN/C(NN)=C1\C=CC=CC1=O |
|  |  | CNc, cNC |
|  |  | CN(C)[C@@H](Cc(cc)cc)C(N)=O |
|  |  | CC(c)(c)O |
|  |  | ccc(cc)-c1ccnc(c)n1nc |
|  |  | Cc1cccc2c1CCO2 |
|  |  | Cc(c)cc(-c)n |
|  |  | CC(=O)NCC(=O)N(C)C |
|  |  | C[C@H](N)C(=O)N(C)C |
|  |  | CC(N)C(=O)N(C)C |
|  |  | C=C/C=C/CCC |
| 138 |  | CCC |
|  |  | C[C@H](O)[C@@H](C)C(C)=O |
|  |  | CC(C)=N |
|  |  | ccc(cc)CN(CC)CC |
|  |  | cC(O)CCCN(C)c |
|  |  | ccc(cc)-c1nc(C)cs1 |
|  |  | cc(c)[C@@H]1CN(CC)C[C@@H]1C |
|  |  | cnn(c(c)-c)c(c)n |
|  |  | CCCNC |
| 807 |  | CC=CC(C)(C)C |
|  |  | cc(c)C(C)(OC)c(c)c |
|  |  | O=[N+]([O-])O |
